# Effect of Iron (III) Oxide Powder on Thermal Conductivity and Diffusivity of Lime Mortar

**DOI:** 10.3390/ma14040998

**Published:** 2021-02-20

**Authors:** Francesc Masdeu, Cristian Carmona, Gabriel Horrach, Joan Muñoz

**Affiliations:** Department of Industrial Engineering and Construction, University of Balearic Islands, Ctra. de Valldemossa km 7.5, E07122 Palma de Mallorca, Spain; cristian.carmona@uib.es (C.C.); gabriel.horrach@uib.es (G.H.); joan.munoz@uib.es (J.M.)

**Keywords:** energy saving with materials, energy storage, thermal conductivity, thermal diffusivity, lime mortar, iron (III) oxide

## Abstract

One of the challenges in construction is the improvement of energy efficiency of buildings. Development of construction materials of low thermal conductivity is a straightforward way to improve heat isolating capability of an enclosure. Lime mortar has a number of advantageous and peculiar properties and was widely used until the “irruption” of Portland cement. Currently, lime mortar is still used in restoration of traditional buildings or, according to the urban regulations, in catalogued constructions. The goal of the present study is the improvement of the heat isolating capability of lime mortars. The strategy of this work is the addition of iron (III) oxide powder, which is one of the possible components forming the cements, to a base lime mortar. The reason to choose Fe_2_O_3_ was two-fold. The first reason is low thermal conductivity of Fe_2_O_3_ compared to lime mortar. The second reason is that the low solubility and small size of iron (III) oxide particles have an effect on the thermal conductivity across the lime particles. The effect of iron (III) oxide powder on the thermal conductivity has been experimentally determined by the hot-box method. It has been found that the insulating capacity and thermal inertia of lime mortar is improved significantly by the addition of Fe_2_O_3_ powder, increasing the energy saving of the enclosure.

## 1. Introduction

In recent years, the increased use of energy from fossil fuels has provoked dramatic climate changes. The greenhouse effect, acid rains, and other phenomena are examples of the consequences of an excessive consumption of this kind of energy. According to the United Nation Environment Program, the energy consumption of buildings represents nearly 40% of the world global energy [1], and around two-thirds of the energy demand in the residential sector is attributed to heating and cooling [2]. The field of construction can assist to mitigate these effects on global warming by improving the performance of construction materials, e.g., increasing heat insulating capability. During recent years, in order to reduce the consumption of energy, great efforts have been devoted in developing low thermal conductivity construction materials and improving the efficiency of materials currently in use [1,3].

One of the potentially favorable materials is the lime mortar. The hardening process of lime is caused by a carbonation reaction [4]. This process requires a long time, especially when compared to Portland cement. However, the use of lime has some advantages, such as strain accommodation (plastic behavior), lower thermal conductivity, or higher breathability, which makes the houses more comfortable [5].

Historically, lime mortar has been widely used around the Mediterranean seaside, mainly as mortar of plaster in vertical walls. The improvement of thermal insulation capacity is especially important for its use as an outer layer of the building enclosure, separating the indoor environment from the outside. From a geological point of view, lime mortar was one of the few binders of high resistance that could be obtained in great abundance in the Mediterranean region. The aim of the present work is to reduce the thermal conductivity or, in other words, to enhance the heat insulating capability of the lime mortar. A common practice to achieve this goal is the addition of filling particles of organic/vegetal origin, such as cork [6], hemp [7,8], olive stone [9], textile waste [10], straw [11], coconut [12], etc. The strategy of the present study is the addition of ceramic submicron particles, whose use and chemical composition is fully compatible with an ecological concept from the point-of-view of generating future, harmless waste for soil and subsoil.

The reasons behind the study of the lime mortar instead of Portland mortar are as follows: (i) better ecological sustainability of lime mortar, since its production requires lower temperatures and less energy [13,14], (ii) lime has a lower thermal conductivity than Portland mortar [15], being a more efficient thermal insulator at the starting point of the study, and (iii) higher indoor comfort provided by lime, since it has a higher breathability [5] and is biocide [16].

The hydraulicity index (HI) that allows one to identify the main chemical components forming the lime cement [17,18,19] is given by:

(1) $$HI=\frac{\left[SiO_{2}\right]+\left[Al_{2}O_{3}\right]+\left[Fe_{2}O_{3}\right]}{\left[CaO\right]+\left[MgO\right]}$$

where the terms in square brackets are the percentages of the five oxides composing the lime. Taking into account that lime cements are composed mostly of calcium or magnesium oxides, there are three candidates that could be selected as an additive to improve the heat insulating capability: SiO_2_, Al_2_O_3_, and Fe_2_O_3_. A comparison of the properties of these candidates shows that iron (III) oxide has the lowest thermal conductivity (λ_Fe2O3_ = 0.58 W/(m·K), λ_SiO2_ = 1.1 W/(m·K), λ_Al2O3_ = 25 W/(m·K)), which is also lower than that of the limestone (λ_limestone_ = 1.3 W/(m·K)) [20,21]. Moreover, iron (III) oxide is an inexpensive mineral and is the seventh most abundant compound in the Earth’s crust [22], which are important factors for a mineral to be used as a construction material.

From the point-of-view of environmental sustainability, iron (III) oxide (hematite) is a component present in farmlands, which is beneficial for plant species. For this reason, the rubbles generated after the stage in the service of buildings would not have a detrimental effect on the environment [23]. Keeping in mind these favorable properties, Fe_2_O_3_ powder has been selected as an additive to improve the thermal efficiency of lime mortar.

The influence of red and black iron oxides addition on the mechanical and physiochemical properties of a concrete was studied by Kishar et al. [24]. A notable positive effect of both red and black iron oxide particles on slump and compressive strength (up to 22–30%) was reported. Recently, Largeau et al. [25] investigated the effect of Fe_2_O_3_ on the strength and workability of a Portland cement concrete. They found that fine Fe_2_O_3_ particles, ca. 200 nm, reduced the porosity and improved compressive strength of concrete for concentrations up to 2.5 wt.%. The present authors are not aware of further research on the effect of iron oxide particles on the thermal properties of lime or Portland mortars.

In this work, the effect of adding small particles of iron (III) oxide on lime mortar has been investigated with the aim of improving the thermal properties of the base lime mortar. It has been found that the insulating capacity and thermal inertia of lime mortar is improved significantly by adding Fe_2_O_3_ powder, increasing the energy saving of the enclosure [26].

## 2. Materials and Methods

### 2.1. Components

Natural hydraulic lime NHL-3.5 Morcem Cal Base 434 CR CSII W0 (Grupo Puma, Malaga, Spain) was selected to use as a base product of the lime mortar. Red iron (III) oxide (Labkem, Barcelona, Spain) of chemical purity higher than 95% was used as the additive.

### 2.2. Components’ Dimensional Characterization

Dimensional characterization of the two components has been carried out using two different techniques. Scanning electronic microscopy (SEM) images, obtained using a Hitachi S-3400N (manufactured by Hitachi Science Systems Ltd., Tokyo, Japan), allowed us to determine characteristic values of particle sizes as well as to visualize the distribution of particles of both Fe_2_O_3_ and lime.

Precise values of grain size and specific surface, measured respectively by means of Beckman Coulter and a Malvern Mastersizer Micro Plus (Malvern Panalytical, Malvern, UK), are shown in Table 1. The range of grain sizes refers to an interval including more than 80% of particle sizes.

### 2.3. Mortar Preparation and Curing

In order to analyze the effect of Fe_2_O_3_ particles on the thermal properties of a lime mortar, five samples with different iron (III) oxide content were prepared. The quantity of water added to the dry mixture was higher as the iron (III) oxide content increases in order to obtain a cement lime mortar of equal workability and elastic consistency, according to the ISO 12439 standard [27]. Table 2 shows the mass and mass fraction of iron (III) oxide powder substituting the lime mortar. The letters LF in the sample notation refer to the mortar components: lime as L and Fe_2_O_3_ as F, while the number (LF-0, LF-5, …, LF-20) denotes the iron (III) oxide mass fraction of each mortar.

**Figure 1:**
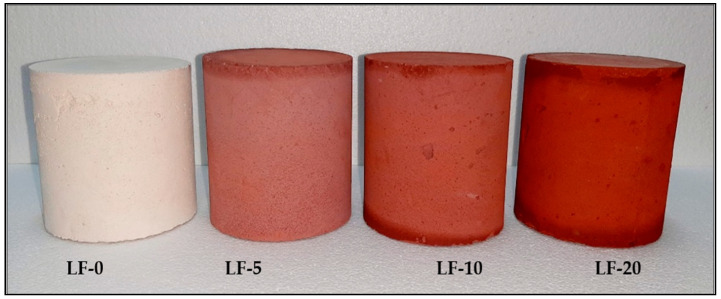
Image of some of the cylindrical samples: LF-0, LF-5, LF-10, and LF-20 (from left to right).

Cylindrical samples 12 cm in height and 10 cm in diameter (Figure 1) were produced using a plastic mold. Curing time was 60 days. The mass of water needed to obtain the optimal mixing increases with iron (III) oxide content since the addition of Fe_2_O_3_ small-size particles increases the specific area, demanding an increasing addition of water to surround the surface of the particles.

### 2.4. Experimental Method

#### 2.4.1. Apparent Density

Apparent density of the studied mortars was calculated from their weight and dimensions of the cylindrical samples [9].

#### 2.4.2. Specific Heat Capacity

Specific heat capacity of mortars was measured using a TA Instruments DSC2920 (manufactured by TA Instruments, New Castle, DE, USA) calorimeter in the modulation mode (MDSC), calibrated with a sapphire sample (error lower than 1%). Measurements were performed at 25 °C using mortar samples weighing around 1.0 mg. Due to their low weight, four different samples were taken from each mortar and tested by MDSC in order to balance out the composition heterogeneity of the mortar.

#### 2.4.3. Thermal Conductivity of Mortars

**Figure 2:**
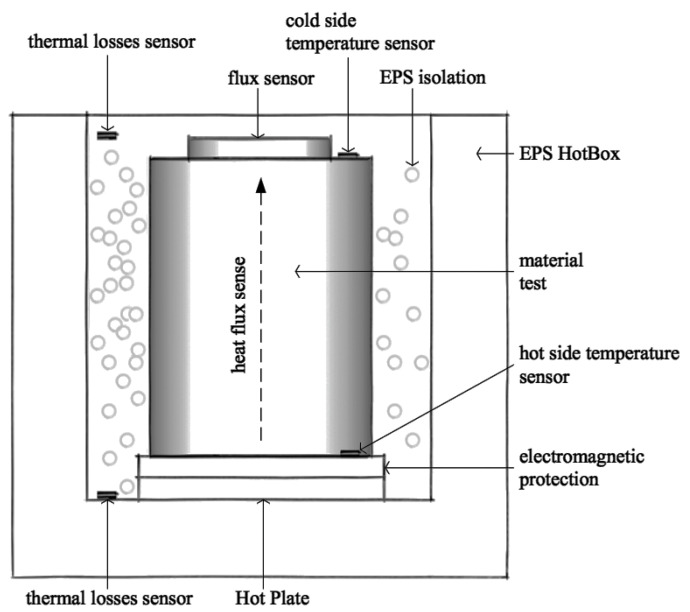
Home-made hot-box design (see text for details).

Thermal conductivity was determined by means of a calibrated hot-box method [28] in a home-made device, shown schematically in Figure 2. The case of the device was fabricated from expanded polystyrene (EPS). The hot plate was placed at the bottom of the case and supported the sample. In the steady state conditions, the hot plate maintains a controlled fixed temperature of the hot side of the sample, equal to 61.2 °C. The heat flux on the cold side of the sample is measured using a HFP01 flux sensor (Hukseflux, Delft, The Netherlands). The temperature is measured on both cold and hot sides of samples by means of thermo-couples. The three parameters obtained in this experiment are temperatures on both hot and cold sides, and the heat flux. The electromagnetic protection serves to distribute homogeneously, over the sample section, with the heat generated by the hot plate. Thermal loss sensors permit controlling the isolating efficiency of the box for an optimal measurement of the heat flow through the sample.

## 3. Results and Discussion

### 3.1. Density and Porosity

The analysis of density and porosity of the sample set provides information crucial for understanding the behavior of thermal conductivity. Theoretical bulk densities were calculated from the bulk density of calcite (2.71 g/cm^3^), which is the main component in lime mortar, and iron (III) oxide (5.26 g/cm^3^) considering their volume fractions for each sample [29]. Porosity was calculated using the theoretical bulk density and apparent density, as 1-(d_apparent_/d_theor_).

**Figure 3:**
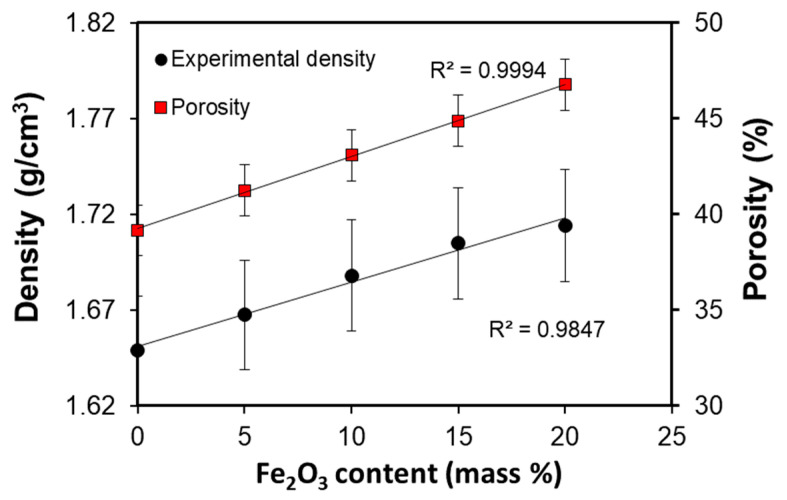
Apparent density and porosity versus Fe_2_O_3_ content of the studied samples.

Apparent density and porosity versus iron (III) oxide content from Table 3 are shown in Figure 3. Both parameters show a linear dependence with the iron (III) oxide content. The pores in mortars are created during the process of curing due to evaporation of water. With the increase of the content of Fe_2_O_3_ submicron particles, the amount of water needed to prepare the mixture increases (see Table 2). Thus, the degree of the porosity generated by releasing water becomes higher.

Thus, the addition of Fe_2_O_3_ results in a moderate increase of density (by ca. 4% for LF-20 compared with the base mortar LF-0) and a more substantial increase of porosity (relative increment around 19% for LF-20). The increase of porosity is known to significantly affect thermal conductivity of the material [30,31,32], which is analyzed in the following section.

### 3.2. Thermal Conductivity

**Figure 4:**
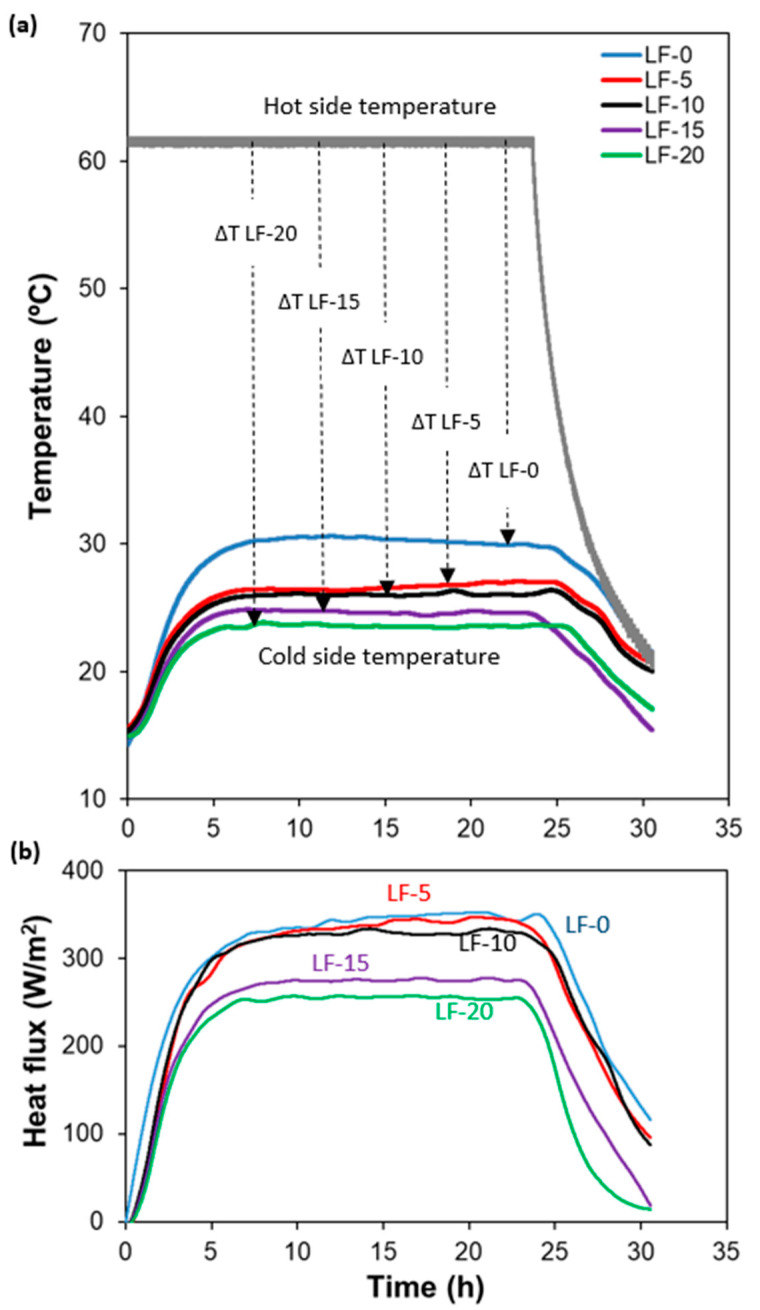
Cold side and hot side temperatures (**a**) and heat flux (**b**) vs. time for samples of lime mortar with a different content of iron (III) oxide.

The kinetics of the heat flux for samples with different Fe_2_O_3_ content is shown in Figure 4. The temperature of the hot plate was set to 61.2 °C at t = 0 and the heating was switched off at t = 23 h. Room temperature was around 15 °C during all tests. According to this experimental protocol, three clearly defined stages are observed in the heat flux kinetics. In the first stage, during the first 8 h, samples heat up from room temperature to an equilibrium value, thus, reaching the steady state regime. During the second stage, between 8 and 23 h, the system is in stationary conditions. After switching off heating of the hot plate at t = 23 h, the samples are cooled down to room temperature.

Figure 4a shows that, in the steady state regime, the temperature difference between the hot and cold sides increases progressively with iron (III) oxide content. Small fluctuations of cold side temperatures observed in this regime could be due to the minor variations of the room temperature during the test. The cold side temperatures of each sample used for the later calculations is an average of measured temperatures in the steady state regime between 8 and 23 h of the test.

More specifically, the values of temperatures at the cold side for the two extreme compositions, LF-0 and LF-20, were 30.5 °C and 23.7 °C, respectively. Taking into account that room temperature during the test was 15 ± 1 °C, these data mean that the increase of temperature for LF-0 and LF-20 are 15.5 °C and 8.7 °C, respectively. Thus, the temperature increase for LF-0 is twice that for LF-20. If the length of all samples was kept similar (less than 2.5% difference), the measured temperatures on the cold side give a first intuitive idea of the different efficiency of thermal isolation of the two materials.

The numerical values of thermal conductivity were calculated using the heat flux through each mortar given by the heat flux-time diagram (Figure 4b), which shows the same tendency as cold side temperature-time dependence. In fact, there exists a direct relation between both parameters, since the heat flux arriving at the cold side of the sample contributes to heating up the material on the cold side. Parameters associated with thermal conductivity are summarized in Table 4. The values of heat flux appearing in Table 4 were determined as an average of results between 8 and 23 h. Experimental thermal conductivity values were obtained from the hot-box measurements at a steady state regime (Table 4). Transmittance, U, was calculated from the measured heat flux, Φ, and the temperature difference between hot and cold sides, ΔT, using the equation obtained from the Fourier’s law of heat conduction [33].

(2) $$U=\frac{\Phi }{\Delta T}$$

The experimental thermal conductivity, λ_exp_, also shown in Table 4, was calculated from transmittance, U, and sample length, L, using the well-known relationship [33].

(3) λ=U·L

The meaning of energy saving, E.S., is the percentage of reduction of heat losses through a wall of a fixed thickness and area, separating two spaces with a certain temperature difference, made of a mortar containing iron (III) oxide compared to the base mortar.

Another meaning of the E.S. is the percentage of power saved by a heating (or cooling) device to keep a certain temperature difference between two spaces separated by a wall of a fixed thickness and area made of a mortar with Fe_2_O_3_ addition compared to the base mortar. Correspondingly, in this work, the E.S. was calculated as the percentage of thermal conductivity reduction taking the value for the LF-0 mortar as a reference.

**Figure 5:**
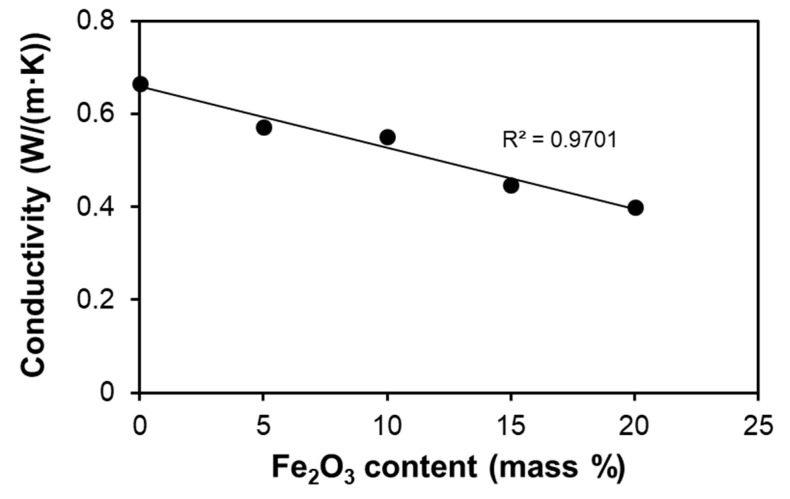
Thermal conductivity dependence with iron (III) oxide content of the lime mortar.

Experimental values of thermal conductivities of different mortars taken from Table 4 are compared in Figure 5. The data show a progressive essentially linear drop of experimental values of thermal conductivity with the increase of Fe_2_O_3_ content.

Two reasons account for the significant thermal conductivity decrease with adding Fe_2_O_3_ powder to the limestone. First, the thermal conductivity of the additive (λ_Fe2O3_ = 0.58 W/(m·K)) [21] is half that of the base material (λ_limestone_ = 1.2 W/(m·K)) [21]. Second, the porosity of mortar increases with the addition of iron (III) oxide, taking the values from 39.2% in LF-0 to 46.8% in LF-20. An equation, which correlates well with the thermal conductivity of the ceramic bodies with the porosity, was proposed by Aivazov and Domashnev [34].

(4) $$\frac{\lambda }{\lambda_{0}}=1-P+n\cdot P^{2}$$

where λ and λ_0_ are the thermal conductivities of a porous and pore-free ceramic bodies, respectively, P is the volume fraction of the pores, and n is a constant. According to Equation (3), which can only be used for a constant bulk material, the thermal conductivity drops with the increasing porosity in the mortar as the iron (III) oxide content raises fulfilling a parabolic function. Therefore, as far as the studied mortar is a combination of two different solids, the thermal conductivity of the bulk material, λ_0_, diminishes in each mortar as the Fe_2_O_3_ content rises.

**Figure 6:**
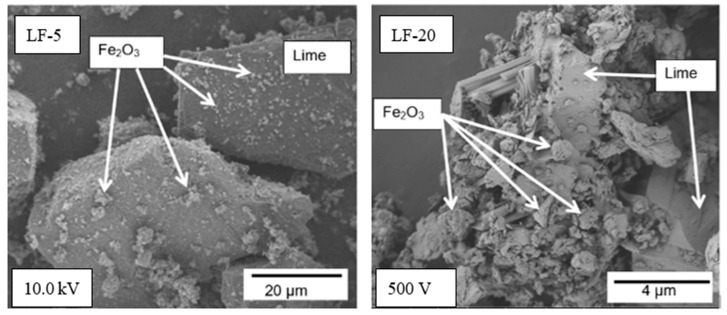
SEM images of the microstructure of mortars with 5% and 20% of Fe_2_O_3_ particles (LF-5 and LF-20, respectively), showing spatial distribution of lime and iron (III) oxide particles.

At the microstructural level, two other reasons for a notable decrease of thermal conductivity with Fe_2_O_3_ additions can be suggested. First, the solubility product constant of the lime cement (Ca(OH)_2_: K_sp_ = 4.68 × 10^−6^) [29] is several orders of magnitude higher than those of iron (III) oxide in both the hydrated or ionic forms in alkali media [35], which are between 4.87 × 10^−17^ and 2.64 × 10^−39^ [29]. Due to extremely low solubility of Fe forms in aqueous media, the number of Fe^3+^ complex ions available to be transported to the neck between particles is very low. In this way, the area of the neck formed between iron (III) oxide particles is small compared to the necks between lime particles. Taking into account that the heat transfer through lime or the Fe_2_O_3_ solid phase is more efficient than through air pores (considering their thermal conductivities [20,21,34]), the reduction of the contact area between solid particles forces the thermal conductivity of the mortar to decrease. Second, scanning electron microscopy (SEM) images of LF-5 and LF-20 (Figure 6) shows that relatively small iron (III) oxide particles stick on the lime particles’ surfaces. This spatial distribution forces lime particles to keep better separated than in the absence of Fe_2_O_3_ fine powder, thus, reducing the contact area between better heat conducting lime particles. A comparison of microstructures of LF-5 and LF-20 mortars in Figure 6 indicates that, with the increase of iron (III) oxide content, lime grains are better separated by small Fe_2_O_3_ particles that stick onto their surfaces.

The overall decrease of the thermal conductivity by the addition of Fe_2_O_3_ powder is due to the combination of the previously mentioned factors: porosity, thermal conductivity of each mortar component, and microstructure.

Apart from the parameters discussed, absorbed water could modify thermal conductivity in porous ceramics [36]. In order to estimate this contribution, the free water content of samples was tested by thermogravimetry. Mass losses due to free water desorption from base lime mortar, LF-0, and the mortar with 20% of Fe_2_O_3_ powder, LF-20, were measured during heating from 20 °C up to 200 °C following standard protocols [37]. In both samples, the losses of water at 120 °C are very similar at around 1.5% (weight). As a consequence, the contribution of free water absorbed by the studied samples is not expected to have a significant effect on the variation of thermal conductivity.

### 3.3. Thermal Diffusivity

Qualitative property of “thermal inertia” was defined by Ng et al. [38] as the ‘property of a material that expresses the degree of slowness with which its temperature reaches that of the environment.’ However, the definition that likely best expresses the effects it causes in an enclosure is the ‘capacity of a material to store heat and to delay its transmission’ due to Ferrari [39]. Then, to keep a constant temperature inside the building when the external temperature changes, the wall’s material should have a thermal inertia as high as possible. One of the parameters characterizing thermal inertia is the thermal diffusivity, a, which can be calculated from thermal conductivity, λ, specific heat capacity, C_e_, and density, d, [40].

(5) $$a=\frac{\lambda }{d\cdot C_{e}}$$

The thermal inertia of the material grows if the thermal diffusivity expressed by Equation (4) decreases.

Specific heat capacity was measured using modulated differential scanning calorimeter (MDSC). Applying a sinusoidal heating rate around a linear temperature permits the measurement of the sample’s heat capacity [41]. The total heat flow, dH/dt, is equivalent to standard differential scanning calorimeter (DSC) at the same average heating rate, and can be calculated using the following equation [41].

(6) $$\frac{\mathrm{dH}}{\mathrm{dt}}=C_{e}\frac{\mathrm{dT}}{\mathrm{dt}}+f\left(T,t\right)$$

where C_e_ is the specific heat capacity, dT/dt is the measured heating rate, C_e_(dT/dt) is the reversing heat flow component of the total heat flow, and f(T,t) is the kinetic component.

**Figure 7:**
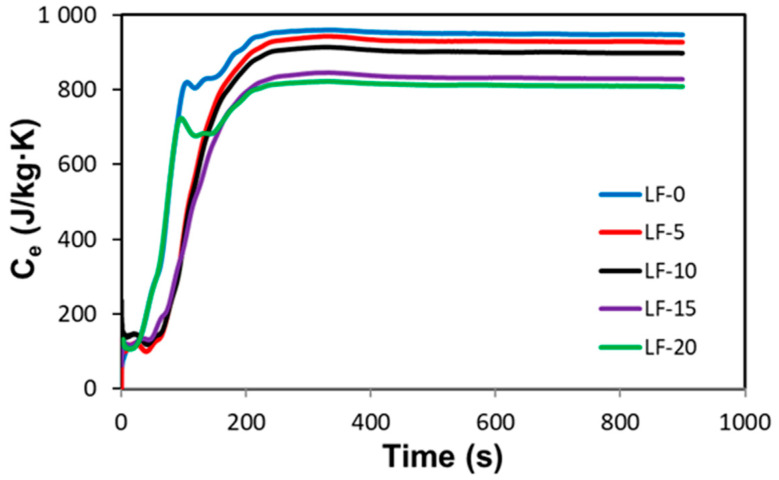
Representative curves of specific heat capacity C_e_ vs. time measured by MDSC for each mortar composition.

Figure 7 shows representative curves of the specific heat capacity, C_e_, evolution with time for each mortar, measured around a linear temperature of 273 K. The values of specific heat capacity for each measurement have been calculated as the average of values between 600 and 900 s, when the C_e_ values of the MDSC measurement is already in a steady state regime.

Table 5 shows the values of specific heat capacity for the measurements made on four different samples of each mortar as well as the average values with standard deviations. Figure 7 and Table 5 indicate clearly that the specific heat capacity of the mortar decreases with Fe_2_O_3_ content. The decrement of specific heat capacity for the LF-20 mortar compared to the base lime mortar LF-0 is 7.6%.

The reason why the specific heat capacity decreases with the addition of Fe_2_O_3_ powder, is that the specific heat capacity of the additive (Fe_2_O_3_: C_e_ = 570 J/(kg·K)) [20] is much lower than that of the base material (lime mortar: C_e_ = 931 J/(kg·K)). It is worth to note that the specific heat capacity of a mixture of solid materials can be calculated using the rule of mixture as the sum of the mass fraction of each component by its specific heat capacity [42]. The corresponding values, shown in Table 5, are in good agreement with experimental data.

Thermal insulation additives for mortars used in building, as cork [6], expanded clay [43] or expanded polystyrene [44], and have a very low density due to their high content of air (high porosity), which makes it difficult to transfer heat through the material. As a consequence of very low density, those insulation materials have an extremely low heat storage capacity per unit volume, which leads to a high thermal diffusivity, or, in other words, a very poor thermal inertia. Contrary to that, the additive used in the current work is heavier than the base mortar. Therefore, the density of the mortar slightly increases by the addition of Fe_2_O_3_. The effects of increasing density and decrease of specific heat capacity on thermal diffusivity are opposite (Equation (4)) and nearly compensate each other, as is shown in Table 6. Hence, the decrease of the thermal diffusivity observed is largely due to the variation of the heat conductivity.

**Figure 8:**
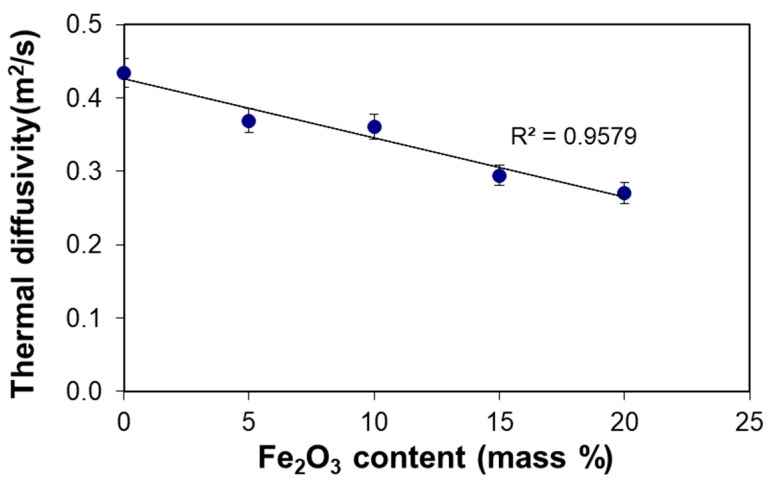
Thermal diffusivity of the lime mortar versus iron (III) oxide content.

The improvement in efficiency related to thermal inertia of LF-20 is over 35% compared to LF-0. The data from Table 6 yield a linear dependence of thermal diffusivity with iron (III) oxide content in the lime mortar (Figure 8).

## 4. Conclusions

Addition of iron (III) oxide is an efficient way to enhance the thermal insulating capacity of a lime mortar. Addition of 20% of Fe_2_O_3_ fine powder to base lime mortar reduces the thermal conductivity by ca. 40% and increases the thermal inertia by ca. 37%, when compared to the base mortar. Thermal conductivity, λ, shows a strong dependence on iron (III) oxide content. The factors improving the thermal properties can be summarized as follows.

1. Thermal conductivity of the used additive, iron (III) oxide, is much lower than that of the lime mortar (base material).
2. The porosity of mortar increases with the addition of Fe_2_O_3_ fine powder from 39% in the base mortar (0% Fe_2_O_3_) to 47% in the mortar containing 20% of Fe_2_O_3_. The thermal conductivity of a porous ceramics drops significantly with the porosity.
3. Due to the extremely low solubility of Fe_2_O_3_ in aqueous media, the area of the neck formed between iron (III) oxide particles is small compared to the necks between lime particles. Therefore, the effective surface of a solid phase able to transfer the heat by conduction diminishes, thus, improving the thermal insulation capability.
4. The use of iron (III) oxide as an additive, which causes an increase of density and a decrease of thermal conductivity compared to base mortar, leads to a significant improvement of the thermal inertia of the resulting mortar.

The future work should consist in studying the influence of Fe_2_O_3_ additions on the mechanical properties of lime mortar and their stability. Once the better thermal efficiency of the new material has been demonstrated, the mechanical strength under compression becomes of a prime importance. Previous works on the addition of iron oxide particles to Portland cement provide positive expectations in this sense.

## 5. Patents

Part of the results of the present article were registered in the following utility model: Masdeu, F.; Muñoz, J.; Carmona, C.; Horrach, G. Mortero de cal termoaislante y su uso en edificación. Spanish Patent ES1222024 U, 2018.

## Figures and Tables

**Table 1:** Grain size and specific surface of lime mortar and iron (III) oxide powders.

Material	Grain Size Range (µm)	Specific Surface (cm^2^/g)
Lime	10–2000	5.7 × 10^3^
Fe_2_O_3_	0.2–0.5	9.4 × 10^4^

**Table 2:** Samples and compositions.

Sample	Component Mass (g)			Fe_2_O_3_ Mass Fraction in Mortar (%)
	Lime Mortar	Fe_2_O_3_	H_2_O	
LF-0	1900	0	400	0
LF-5	1805	95	412	5
LF-10	1710	190	436	10
LF-15	1615	285	500	15
LF-20	1520	380	545	20

**Table 3:** Bulk and apparent densities and porosity of the samples with different iron (III) oxide content.

Sample	Fe_2_O_3_ Content (mass %)	Theoretical Bulk Density (g/cm^3^)	Apparent Density (g/cm^3^)	Porosity (%)
LF-0	0	2.71 ± 0.02	1.65 ± 0.03	39.2 ± 0.9
LF-5	5	2.84 ± 0.02	1.67 ± 0.03	41.2 ± 0.9
LF-10	10	2.97 ± 0.02	1.69 ± 0.03	43.1 ± 1.0
LF-15	15	3.09 ± 0.02	1.70 ± 0.03	44.9 ± 1.0
LF-20	20	3.22 ± 0.02	1.71 ± 0.03	46.8 ± 1.1

**Table 4:** Experimental thermal conductivity and energy saving of the samples of lime mortar with different iron (III) oxide content.

Sample	T_H_ (°C)	T_C_ (°C)	ΔT (K)	Φ (W/m^2^)	U (W/m^2·^K)	L (m)	λ_exp_ (W/m·K)	E.S. (%)
LF-0	61.2	30.5	30.7	166.2	5.42	0.123	0.67 ± 0.01	0.0
LF-5	61.2	26.4	34.8	165.6	4.76	0.120	0.57 ± 0.01	14.3 ± 0.4
LF-10	61.2	26.0	35.2	160.5	4.56	0.121	0.55 ± 0.01	17.2 ± 0.5
LF-15	61.2	24.7	36.5	134.0	3.67	0.122	0.45 ± 0.01	32.8 ± 1.0
LF-20	61.2	23.7	37.5	124.4	3.32	0.120	0.40 ± 0.01	40.1 ± 1.2

T_H_: hot side temperature. T_C_: cold side temperature. ΔT: difference between hot and cold sides. Φ: heat flux. U: transmittance. L: sample length. λ_exp_: experimental thermal conductivity. E.S.: energy saving.

**Table 5:** Specific heat capacity values, C_e_, calculated from MDSC measurements.

Sample	Specific Heat Capacity, C_e_ (J/(kg·K))							Decrement ^1^ (%)
	Test 1	Test 2	Test 3	Test 4	Average	Standard Dev.	Rule of Mixture	
LF-0	922	934	926	939	931	8	931 ± 3	0.0
LF-5	933	920	929	925	927	6	913 ± 3	0.43 ± 0.01
LF-10	898	912	900	917	907	9	895 ± 3	2.58 ± 0.03
LF-15	887	896	883	902	892	9	877 ± 3	4.19 ± 0.05
LF-20	866	852	875	847	860	13	859 ± 3	7.63 ± 0.13

^1^ Decrement calculated using LF-0 as a reference.

**Table 6:** Thermal diffusivity of the samples of lime mortar with different iron (III) oxide content calculated using Equation (3) from experimental values of thermal conductivity, λ, specific heat capacity, C_e_, and density, d.

Sample	Density ^1^, d (kg/m^3^)	Specific Heat Capacity, C_e_ (kJ/(kg·K))	d·C_e_ (kJ/m^3^·K)	Thermal Conductivity, λ (kW/(m·K))	Diffusivity, a (m^2^/s)	Improvement ^2^ (%)
LF-0	1649	0.931	1535	666	0.434 ± 0.020	0.0
LF-5	1667	0.927	1545	571	0.369 ± 0.016	14.9 ± 1.3
LF-10	1688	0.907	1531	552	0.360 ± 0.017	17.0 ± 1.6
LF-15	1705	0.892	1521	448	0.294 ± 0.014	32.2 ± 3.0
LF-20	1714	0.860	1474	399	0.271 ± 0.014	37.6 ± 3.7

^1^ The values of density in Table 6 are those shown in Table 3 as apparent density. ^2^ Improvement calculated using LF-0 as a reference.

## Data Availability

Data Sharing is not applicable.
